# Supplementary figures and images for: Reproducibility of SNV-calling in multiple sequencing runs from single tumors
Source: PeerJ. 2016 Jan 4;4:e1508. doi: 10.7717/peerj.1508 (PMC4741064; doi:10.7717/peerj.1508)

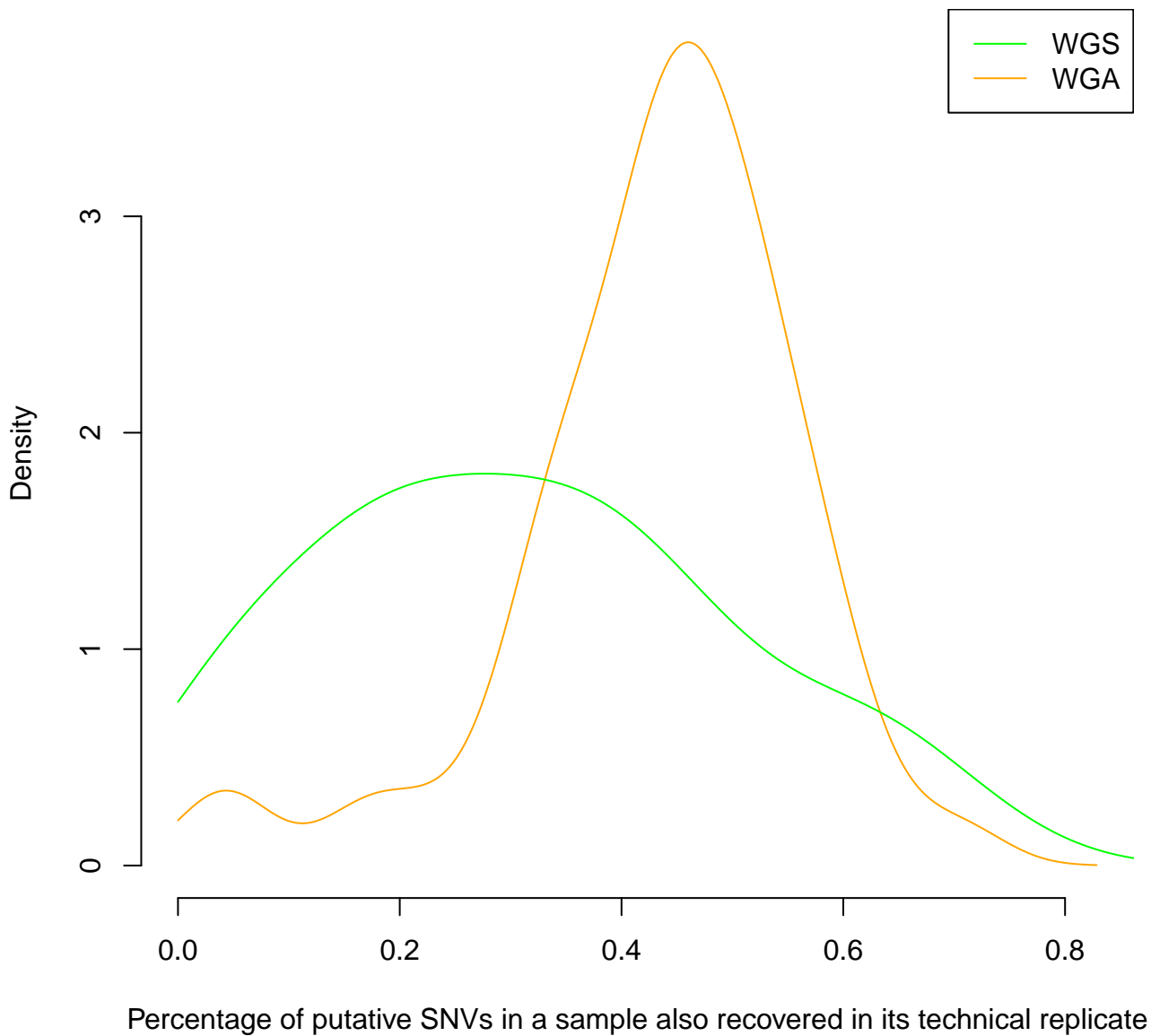

Supplement: Figure S1 — For each pair of replicates (WGS and WGA), we looked at the percentage of WGS SNVs that were recovered in the WGA sample (about one half, in green), and the percentage of the WGA SNVs that were recovered in the WGS sample (about one third, in orange). The WGS distribution is higher and narrower, showing that the WGS samples overall have a higher percentage overlap than the WGA samples, and less range in this parameter. [file peerj-04-1508-s001.pdf]

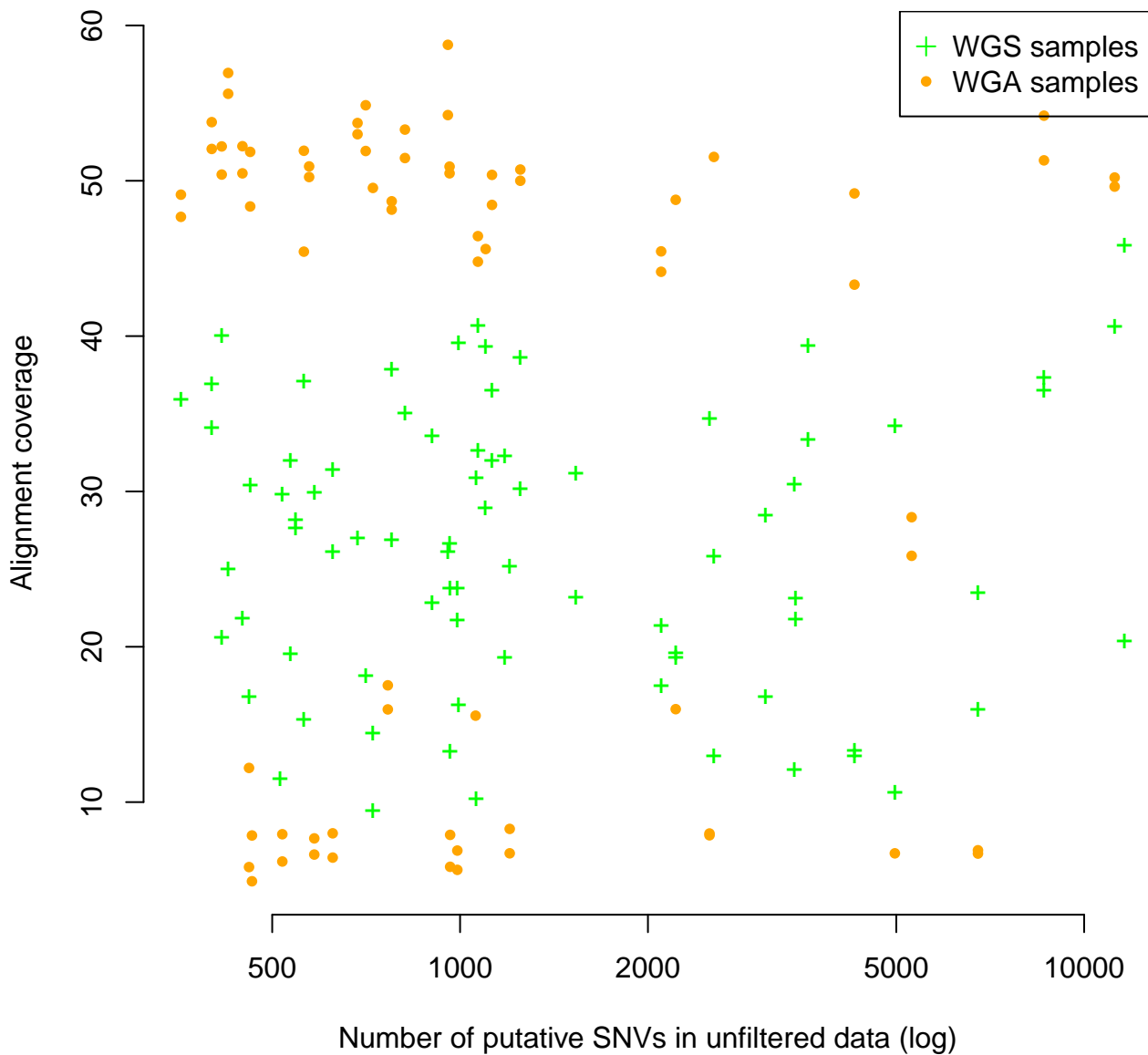

Supplement: Figure S2 — The number of SNVs called in a sample does not correlate with the coverage of that sample (Spearman ρ = − 0.13, S = 671817, P = 0.12). This is shown by the consistent variability along the x-axis at each level of overage (shown on the y-axis). The separation of the two experimental condition on the y-axis is not relevant to this measure. [file peerj-04-1508-s002.pdf]

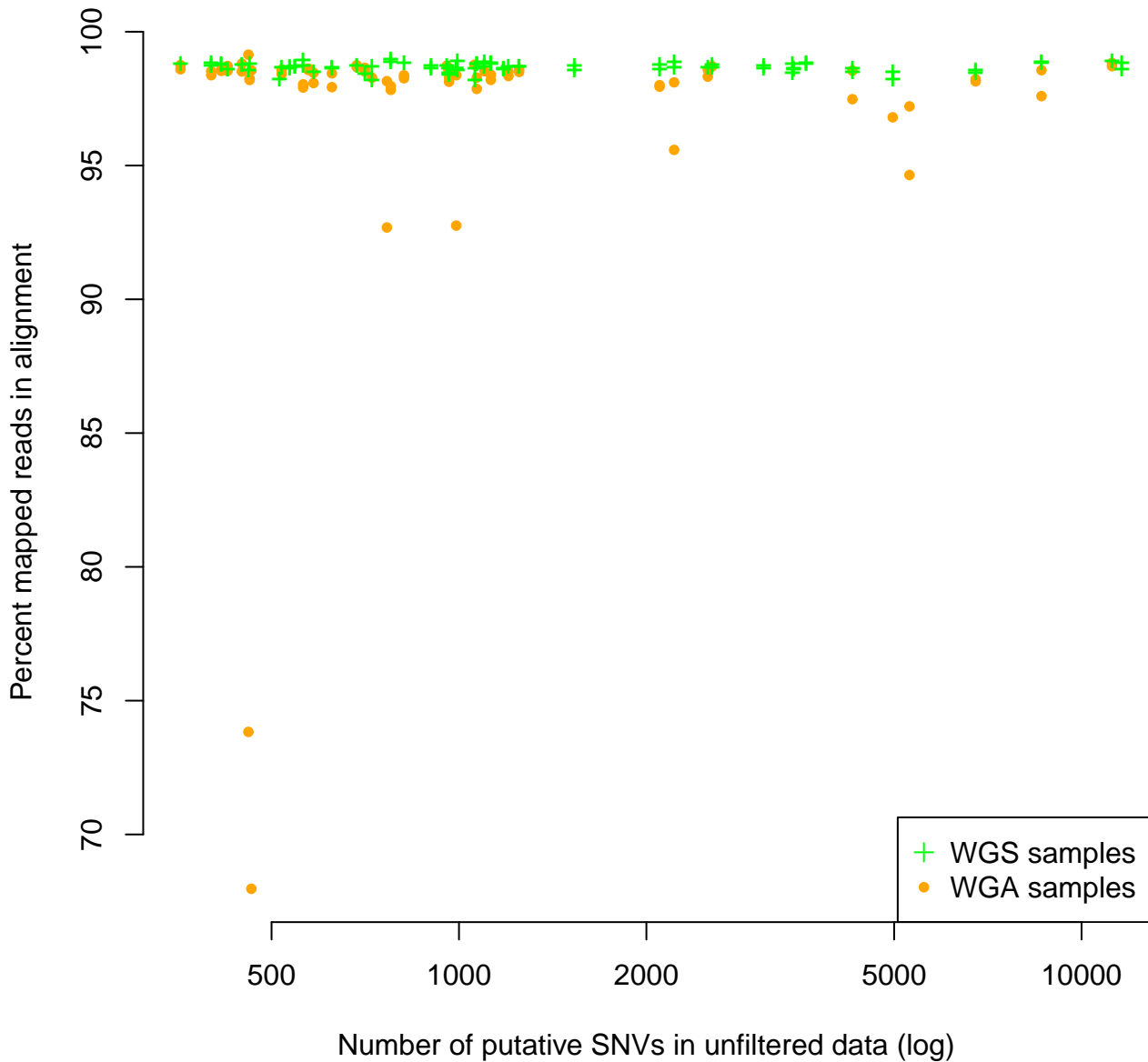

Supplement: Figure S3 — The number of SNVs called in a sample does not correlate with the percentage of mapped reads in the alignment of that sample (Spearman ρ = − 0.068, S = 637326, P = 0.41). [file peerj-04-1508-s003.pdf]
