# Supplementary material for: Reproducibility of SNV-calling in multiple sequencing runs from single tumors
Source: PeerJ. 2016 Jan 4;4:e1508. doi: 10.7717/peerj.1508 (PMC4741064; doi:10.7717/peerj.1508)
Supplement: Table S1 — This table shows the software packages we used in data processing, what we used each piece of software for, and the command associated with the use of it. The rows are in order of use. [file peerj-04-1508-s004.pdf]

<table>
<tr><th>software</th><th>purpose</th><th>command</th></tr>
<tr><td>picard</td><td>regenerate fastq files from BAM file aligned to hg18</td><td>java -d64 -Xmx4g -jar SamToFastq.jar I=$pfx.bam F=$pfx.1.fastq F2=$pfx.2.fastq 2>&1</td></tr>
<tr><td>bwa</td><td>align fastq files to hg19</td><td>bwa aln -q 30 -t 8 $hgReference $fastq > $fastq.aln.sai</td></tr>
<tr><td>bwa, samtools</td><td>convert aligned fastq files into new BAM file</td><td>bwa sampe -a 600 -P -r "$RG" $hgReference $fastq1.aln.sai $fastq2.aln.sai $fastq1 $fastq2 | samtools view -bSh -o $outprefix.bam -</td></tr>
<tr><td>samtools</td><td>sort and index new BAM file</td><td>samtools sort -@ 16 $outprefix.bam $outprefix.sorted 2, samtools index $outprefix.sorted.bam 2</td></tr>
<tr><td>samtools</td><td>remove duplicate reads from BAM files</td><td>samtools rmdup ../$tumorpfx/$tumorpfx.out.sorted.bam $tumorpfx.dedup.bam</td></tr>
<tr><td>GATK</td><td>indel realignment</td><td>java -d64 -jar $gatkJar -R $hgReference -T IndelRealigner -rf BadCigar -I $tumorpfx.dedup.bam -known $G1000.Mills -known $G1000.Phase1.Indels -targetIntervals $tumorpfx.intervals -o $tumorpfx.realn.bam</td></tr>
<tr><td>GATK</td><td>base recalibration</td><td>java -d64 -jar $gatkJar -nct 8 -T BaseRecalibrator -rf BadCigar -I $tumorpfx.realn.bam -R $hgReference -knownSites $dbSNP -o $tumorpfx.recal.grp</td></tr>
<tr><td>samtools</td><td>index recalibrated BAM file</td><td>samtools index $tumorpfx.realn.recal.bam</td></tr>
<tr><td>SomaticSniper</td><td>call somatic mutations, generate VCF</td><td>bam-somaticsniper -q 40 -Q 40 -J -s 0.001 -F vcf -f $hgReference $tumorbam $normalbam $tumorpfx.SS.vcf</td></tr>
</table>
